# Applications of Liquid Biopsies in Non-Small-Cell Lung Cancer

**DOI:** 10.3390/diagnostics12081799

**Published:** 2022-07-25

**Authors:** Martin Pesta, Dattatrya Shetti, Vlastimil Kulda, Tereza Knizkova, Katerina Houfkova, Mahyar Sharif Bagheri, Martin Svaton, Jiri Polivka

**Affiliations:** 1 Department of Biology, Faculty of Medicine in Pilsen, Charles University, Alej Svobody 1655/76, 323 00 Plzen, Czech Republic; dattakapilshetti@gmail.com (D.S.); teraza.knizkova@lfp.cuni.cz (T.K.); katerina.houfkova@lfp.cuni.cz (K.H.); 2 Department of Medical Chemistry and Biochemistry, Faculty of Medicine in Pilsen, Charles University, Karlovarska 48, 301 66 Plzen, Czech Republic; vlastimil.kulda@lfp.cuni.cz; 3 Department of Histology, Faculty of Medicine in Pilsen, Charles University, Karlovarska 48, 301 66 Plzen, Czech Republic; mahyar.sharif@lfp.cuni.cz (M.S.B.); jiri.polivka@lfp.cuni.cz (J.P.); 4 Department of Pneumology and Phthisiology, Faculty of Medicine in Pilsen, Charles University, University Hospital in Pilsen, E. Benese 13, 301 00 Plzen, Czech Republic; svaton@fnplzen.cz

**Keywords:** lung cancer, NSCLC, liquid biopsy, ctDNA, CTC, *EGFR*, *KRAS*, *ALK*

## Abstract

The concept of liquid biopsy as an analysis tool for non-solid tissue carried out for the purpose of providing information about solid tumors was introduced approximately 20 years ago. Additional to the detection of circulating tumor cells (CTCs), the liquid biopsy approach quickly included the analysis of circulating tumor DNA (ctDNA) and other tumor-derived markers such as circulating cell-free RNA or extracellular vesicles. Liquid biopsy is a non-invasive technique for detecting multiple cancer-associated biomarkers that is easy to obtain and can reflect the characteristics of the entire tumor mass. Currently, ctDNA is the key component of the liquid biopsy approach from the point of view of the prognosis assessment, prediction, and monitoring of the treatment of non-small-cell lung cancer (NSCLC) patients. ctDNA in NSCLC patients carries variants or rearrangements that drive carcinogenesis, such as those in *EGFR*, *KRAS*, *ALK*, or *ROS1*. Due to advances in pharmacology, these variants are the subject of targeted therapy. Therefore, the detection of these variants has gained attention in clinical medicine. Recently, methods based on qPCR (ddPCR, BEAMing) and next-generation sequencing (NGS) are the most effective approaches for ctDNA analysis. This review addresses various aspects of the use of liquid biopsy with an emphasis on ctDNA as a biomarker in NSCLC patients.

## 1. Introduction

Lung cancer is recognized as the leading cause of cancer deaths, killing 1.8 million and being diagnosed in 2.2 million people worldwide in 2020 [1]. Europe accounts for 20% of total lung cancer mortalities, with a 5-year survival rate of 11.2% for men and 13.9% for women [2]. Based on the tissue morphology, lung cancer can be divided into two major types: small-cell lung cancer (SCLC), which is the aggressive type of lung cancer, and non-small-cell lung cancer (NSCLC), which shows a slower progression [3]. Surgery remains the first treatment option for early-stage NSCLC patients nowadays. However, after an effective resection, 30–55% of patients still have a possibility of recurrence and may die from the disease [4]. Hence, in this scenario, for the precise prediction of the treatment outcome, disease prognosis, and disease recurrence, specific biomarkers are needed.

The gold standard for the assessment of histological type and the prediction of treatment response has been tissue biopsies, histopathology analysis, and tissue genotyping. Tissue biopsy is a commonly utilized technique; however, despite its irreplaceability, it has some limitations. It involves tissue sampling from a specific place; thus, it cannot be conducted on a regular basis, and it does not reflect the entire malignancy. Liquid biopsy based on the analysis of peripheral blood is an approach that partially overcomes the above-mentioned limitations. The key advantage of liquid biopsy is that it is a non-invasive technique for detecting multiple cancer-associated biomarkers that is easy to obtain and can reflect characteristics of the entire tumor malignancy [5].

The concept of liquid biopsy as an analysis of non-solid tissue for the purpose of providing information about solid tumors was introduced approximately 20 years ago. The concept was established for the analysis of circulating tumor cells (CTCs) [6] and quickly expanded to include circulating tumor DNA (ctDNA) [7] and other tumor-derived markers such as circulating cell-free RNA (noncoding and messenger) [8,9] and extracellular vesicles [10]. However, circulating nucleic acids in plasma or serum (CNAPS) in cancer have been investigated since the 1990s [11,12]. The liquid biopsy concept can also be applied to other body fluids such as cerebrospinal fluid (CSF) or urine.

CTCs and ctDNA are two key components of the liquid biopsy approach from the point of view of the prognosis assessment, prediction, and monitoring of the treatment. The availability of new targeted therapies made CTCs and ctDNA a very attractive and active field of research. The advantages and disadvantages of these two approaches have been reviewed by Calabuig-Fariñas et al. [13]. CTC analysis provides comprehensive information about the phenotype (gene expression and protein analysis) and genotype (analysis of DNA changes) of cancer cells circulating in the bloodstream and allows in vitro studies (analysis of drug resistance). CTCs reflect metastasis initiation and help monitor treatment efficacy [14]. The use of ctDNA is promising for characterizing tumor molecular alterations and identifying variants associated with drug resistance. ctDNA in lung cancer patients carries variants that drive carcinogenesis, such as those in the epidermal growth factor receptor (*EGFR*), Kirsten rat sarcoma virus (*KRAS*), tumor suppressor gene *TP53*, anaplastic lymphoma kinase (*ALK*), reactive oxygen species 1 (*ROS1*), and epigenetic changes such as DNA methylation. The analysis of ctDNA reflects the intratumoral heterogeneity as well as the tumor load [15]. Nevertheless, numerous issues need to be addressed, including sensitivity and reproducibility.

Due to the low concentration of ctDNA in liquid biopsy, sensitive DNA detection techniques are needed, such as digital droplet polymerase chain reaction (ddPCR) and next-generation sequencing (NGS) to detect somatic variants in fragmented circulating cell-free DNA (cfDNA) present at low concentrations and a broad spectrum of variants in ctDNA molecules. In general, the sensitivity of ddPCR is higher than that of NGS [16].

In comparison to CTC detection, the cost of assessment in ctDNA detection is steadily decreasing. This is due to the fact that DNA sequencing or the assessment of particular variants is performed by using techniques that also have applications in other areas of biology and medicine. However, the price of ctDNA analysis is difficult to determine for individual methods, as it is influenced by a number of factors, especially the number of examinations performed by a certified laboratory. In general, digital PCR-based methods are cheaper than NGS-based ones. The economic impact of liquid biopsies in cancer management was reviewed by Ijzerman et al. [17].

According to recent studies, oncological patients exhibit higher levels of cfDNA than healthy individuals. The half-life of cfDNA is 15 min; however, due to the constant shedding, overall concentrations may remain quite stable and even increase with higher tumor grades [18]. Current studies recommend plasma instead of serum as a suitable biological sample to analyze cfDNA or ctDNA [19]. ctDNA enables serial tumor monitoring over time, including the identification of nucleotide variants, epigenetic changes, and the capture of intratumoral heterogeneity [19,20].

However, there are some limitations. One of the reasons for possible discordance between the cfDNA genotyping and tumor/metastasis tissue variants is the phenomenon of clonal hematopoiesis resulting in the overrepresentation of blood cells from a single clone [21]. Hematopoietic cells are the primary source of cfDNA. During aging, white blood cells can acquire somatic variants, which offers a selective growth advantage. Due to the process of clonal expansion, such variants can be amplified to a detectable level. The spectrum of variants detected in cfDNA originating from clonal hematopoiesis can overlap with variants in ctDNA. A large pan-cancer study on over 10,000 patients using parallel sequencing comparing plasma and white blood cells showed that 14% of plasma cfDNA contained clonal hematopoiesis variants. Methods to distinguish between cfDNA variants derived from clonal hematopoiesis and tumor-specific variants are currently being developed [22].

**Figure 1:**
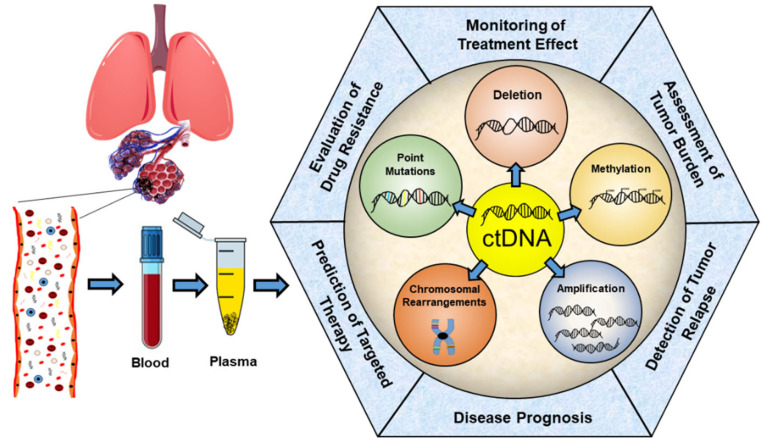
Current research areas of ctDNA application in non-small-cell lung cancer management.

From a clinical perspective, the assessment of variants in ctDNA can provide valuable information complementary to tissue sequencing and offer new opportunities for the prediction of the response to targeted therapy and the monitoring of disease recurrence, but also for diagnosis and prognosis [23], as summarized in Figure 1.

The main focus of this review is on the applications of ctDNA as a liquid biomarker in NSCLC patients. We set out the major steps in oncology management which could profit from ctDNA analysis and discuss the studies focused on these applications.

## 2. ctDNA—Properties and Analysis

While the first recorded detection of CTCs occurred in 1869 (Thomas R. Ashworth) [24], the presence of DNA in blood plasma was initially described in 1948 by Mandel and Metais [25]. The fraction of cell-free DNA (cfDNA) of tumor origin was later termed circulating tumor DNA (ctDNA). It took decades to show that tumor-specific genomic markers could be traced in DNA isolated from the blood plasma of patients with different solid tumors. The first tumor-specific sequences detected in the blood of patients with cancer were found in the mutated DNA of RAS genes. The presence of mutated KRAS was detected for the first time in the blood of patients with pancreatic cancer in 1994 by Sorenson et al. [26].

Despite the progress in the field of cfDNA research in the last decades, the mechanism controlling how DNA enters the bloodstream is not clearly understood yet. However, there is a consensus that the primary origin of cfDNA is a release of DNA from apoptotic and necrotic cells; another process involved can be active secretion as a cargo of exosomes. cfDNA contains fragments usually of 167 bp in length, corresponding to the length of DNA wrapped around a nucleosome, resulting from the cleavage of DNA by a caspase-dependent endonuclease (CAD). cfDNA is present in blood plasma at a concentration between 1 and 100 ng/mL and its half-life is estimated to be from a few minutes to a couple of hours [27].

It was found that ctDNA is more fragmented than cfDNA. Furthermore, ctDNA contains a broader range of lengths of DNA fragments. There is a much higher fraction of <100 bp fragments with a 10 bp periodicity [28], but there are also longer fragments originating from necrotic cells [29].

Recently, methods based on qPCR (ddPCR, BEAMing) and NGS are the most effective approaches for ctDNA analysis. Although ddPCR and BEAMing have high sensitivity, they can only detect known variants using specific probes, leaving the possibility of identifying a large pool of unknown variants. NGS-based methods targeted at loci of interest such as NGS assays, CAPP-Seq, and whole-exome sequencing (WES) allow the detection not only of known variants but also of new variants. While the limit of the detection of PCR-based methods is 0.001–0.01%, the NGS techniques are not so sensitive (0.1–1%). Cancer Personalized Profiling by Deep Sequencing (CAPP-Seq) is an ultra-sensitive assay based on deep sequencing; however, the assessment is much more time-consuming (5 days) and the cost is much higher [30].

All methods mentioned above are already available on the market. To perform ddPCR, the QX200 Droplet Digital PCR System (Biorad, CA, USA) is being widely used. OncoBEAM™ technology is being offered by Sysmex Inostics (Hamburg, Germany). BEAMing (Bead, Emulsion, Amplification, and Magnetics) is a digital PCR method that combines emulsion PCR and flow cytometry to identify and quantify specific sequences of DNA. TruSight Oncology ctDNA (Illumina, San Diego, CA, USA) is a pan-cancer NGS assay that enables comprehensive genomic profiling from blood plasma.

## 3. Prediction of Response to Targeted Therapy

Molecular profiling becomes much more important for revealing genetic variants. Knowing genetic variants is a vital step towards effectively utilizing targeted therapy and creating customized cancer treatment [31,32]. In terms of effectivity (biology, economy), ctDNA analysis is the best liquid biopsy approach to detect targetable variants. The American Society of Clinical Oncology (ASCO) and the National Comprehensive Cancer Network (NCCN) suggested the variants profiling of key driver genes in tissue such as *EGFR*, *KRAS*, *BRAF*, *ALK*, and *ROS1* as a biomarker to predict targeted therapy for patients with advanced NSCLC [33,34]. Recently, the European Medicines Agency (EMA) approved the ctDNA-based detection of variants and phenotypic characterization in onco-driver genes, particularly to predict NSCLC treatment outcomes, and ASCO has included it in its own guidelines [35,36].

### 3.1. EGFR Inhibitors

The EGFR-mediated signaling pathway is crucial for NSCLC cancer cell survival and progression [37,38]. Exon 19 deletions and the L858R point variant in exon 21, which are tyrosine kinase inhibitor (TKI) sensitive, account for 90% of *EGFR* gene changes. The remaining 10% of all variants, such as the T790M variant on exon 20 of *EGFR*, are linked to treatment resistance [39,40]. The activating EGFR variants were first discovered in 2004, and patients with these variants had a better outcome after conventional treatment, indicating that activating *EGFR* variants could be used as a target for personalized treatment [41].

Currently, three generations of EGFR-TKIs are now used for therapy. Erlotinib, gefitinib, and icotinib are first-generation TKIs that bind irreversibly to ATP binding sites, impeding the downstream pathway. Second-generation TKIs (afatinib and dacomitinib) block ErbB3 transphosphorylation by covalent binding to ErbB3 homodimers and heterodimers, providing an alternative therapy for patients resistant to first-generation TKIs. However, the T790M *EGFR* variant is the main cause of resistance to first- and second-generation TKIs in around 50% of patients. Recently, third-generation TKIs (osimertinib, rociletinib, olmutinib, and lazertinib) have shown a much greater affinity with T790M [42]. According to a meta-analysis of 5005 *EGFR*-mutated NSCLC patients, third-generation TKI (osimertinib) appeared to have the highest probability of being the most effective first-line treatment [43]. Unfortunately, tumors develop resistance to these third-generation drugs; hence, researchers are actively investigating novel targeted medicines.

*EGFR* variants proved to be a conventional predictive marker for selecting first-line EGFR-TKI treatment for NSCLC patients with high-grade carcinomas. The detection of *EGFR* variants in tissues has obvious limitations, which is why ctDNA is a good choice of an alternative approach to the detection of *EGFR* variants [44]. In a study by Mok et al. in 2015, the detection of *EGFR*-activating variants in ctDNA was sensitive (75%) and highly specific (96%), with high concordance between matched blood-based and tumor tissue samples (88%), showing that ctDNA analysis may have utility in clinical practice as a predictor of clinical outcomes [45].

Clonal architecture allowing the accumulation of somatic variants is an important feature of cancer biology and plays a prominent role in disease relapse and therapy resistance. A ctDNA approach was established to understand how the clonal architecture of tumors affects advanced-stage cancers. In the ctDNA of IIIB–IV NSCLC patients, target-capture deep sequencing identified *EGFR* as a dominant clone, indicating that *EGFR* could be a prominent marker of EGFR TKI resistance [46].

Liquid biopsy has recently made it feasible to detect the T790M variant in ctDNA and predict resistance to TKIs. Plasma samples of NSCLC patients with an EGFR-activating variant revealed that the T790M variant occurs during treatment with gefitinib, erlotinib, and afatinib, showing a shared mechanism of resistance to first- and second-generation TKIs [47]. A new molecular barcode technique identified other *PIK3CA*, *TP53*, *KRAS*, and *MAP2K1* variants in EGFR TKI-resistant patients, demonstrating that the resistance mechanism involves not just *EGFR* T790M but also other variants in lung cancer patients [48]. An NGS-based study in Chinese patients exhibited different levels of plasma in the *EGFR* T790M variant. During the first-generation EGFR TKI treatment, the *EGFR* T790M variant appeared, disappeared, and reappeared, indicating spatial and temporal diversity due to competitive evolution between different tumor clones. Furthermore, two novel putative drug resistance-associated variants, *EGFR* V769M and *KRAS* A11V, have been found and require further study [49]. Romero et al. performed ddPCR on plasma samples from osimertinib-resistant patients and discovered that roughly 20–30% of instances had a C797S variant observed together with a *PIK3CA* variant, which could be involved in the resistance mechanism [50].

Ishii et al. used CAPP-Seq to analyze the ctDNA of afatinib-treated *EGFR* T790M-positive patients who were resistant to third-generation EGFR-TKI osimertinib. The sequencing data revealed that the C797S variant is responsible for osimertinib [51].

Plasma genotyping can identify different molecular patterns responsible for resistance, suggesting that the use of liquid biopsy is a valuable approach in the detection of resistance-causing variants.

### 3.2. ALK/ROS Inhibitors

The oncogenic activation of anaplastic lymphoma kinase (ALK) in tumors is triggered by forming either a fusion gene or point variants. Patients with activated *ALK* oncogene experience considerable clinical benefits from ALK inhibitors (ALKi) [52,53,54]. Chromosome rearrangements affecting the gene that encodes for ALK have been observed in 3–8% of lung adenocarcinoma patients [55]. The detection of fusion genes performed at the DNA level by PCR-based techniques is limited to the most common fusions with primers or probes close to recurrent breakpoints. Furthermore, NGS methods performed at the DNA level have some pitfalls. Large introns can affect performance, and moreover, there is no discrimination between expressed and unexpressed gene fusions [56].

Several next-generation ALK inhibitors have been authorized by the US Food and Drug Administration (FDA); however, their sensitivity profiles for ALK kinase domain variants differ. This indicates that genomic profiling following the failure of first-line ALKi is essential in NSCLC patients [57,58]. Meta-analysis data showed that crizotinib, ceritinib, alectinib, and brigatinib improved progression-free survival (PFS), whereas alectinib improved overall survival (OS) in ALK-positive NSCLC patients [59].

Although ALK-TKIs have an overall survival record of 81 months, resistance is unavoidable [57]. Crizotinib is the first effective TKI to demonstrate improved effectiveness in NSCLC patients with *ALK* rearrangement. However, disease progression is observed after 9–10 months due to acquired resistance [60]. Acquired resistance to ALK TKIs has been related to both “on-target” genomic changes, such as variants in the ALK tyrosine kinase domain (KD), and the amplification of the *ALK* fusion, as well as the activation of bypass signaling networks [61,62].

Comprehensive genomic profiling (CGP) is a next-generation sequencing-based technique that identified canonical and noncanonical fusion *ALK* rearrangements in patients who responded to ALK inhibitor crizotinib for a long time [63]. McCoach et al. performed a study on ALK-positive patients that aimed to identify fusion partners using Guardant360 (G360; Guardant Health), an NGS-based test conducted on ctDNA. The most common ALK fusion was the one with *EML4* (85.4%), followed by *STRN* (6%), and *KCNQ*, *KLC1*, *KIF5B*, *PPM1B*, and *TGF* (totaling 8.3%) [64].

A capture-based targeted sequencing panel detected crizotinib-resistant variants and quantified *ALK* missense variants (*ALK* L1152R, *ALK* I1171T, and *ALK* L1196M) [65]. Alongside the detection of *ALK* variants, *KRAS* variants have also been found. A study by Bordi et al. revealed that *ALK* and *KRAS* variants are responsible for crizotinib resistance and that *ALK* variants can be identified in plasma and monitored as a response parameter [66]. Several studies established ROS1-positive and ALK-positive patients as separate entities, despite biochemical and clinicopathological similarities. Dagogo-Jack et al. discovered seven distinct ROS 1 fusion partners, where *ROS1* fusing with *CD74* belongs to the most common fusion in ROS1-positive NSCLC patients relapsing after crizotinib [67].

Evidence is scarce on the clinical relevance of *ALK/ROS1* variants in TKI-resistant NSCLC patients. In TKI-failure patients, a targeted amplicon-based assay revealed 22% of patients with *ALK* fusions and 30% with the *ROS1* G2032R variant. The amplicon-based sequencing offers clinically relevant data on *ALK/ROS1* fusions in TKI-resistant heterogeneity, supporting their significance in sequential treatment selection [68]. However, the detection of *ALK/ROS1* rearrangements in ctDNA for the prediction of treatment is still not part of ASCO guidelines.

Recently, Horn et al. researched the efficacy of powerful second-generation ALK TKI (ensartinib) by analyzing ctDNA. Genetic changes prior to ensartinib treatment were detected in 74% of patients, with *EML4-ALK* fusion being the most prevalent. When compared to individuals with an *EML4-ALK* variation 3 (V3) fusion, those with a detectable *EML4-ALK* variation 1 (V1) fusion showed a better response to ensartinib [69]. This shows that plasma genotyping is a promising method to identify variants and fusions responsible for resistance to therapy.

### 3.3. RET Inhibitors

The rearranged during transfection (*RET*) proto-oncogene codes for a receptor tyrosine kinase (RTK). Chromosomal rearrangements or point variants are responsible for the RET proto-oncogene variant, which activates the receptor tyrosine kinase in NSCLC patients. Variants in the *RET* gene can be found in about 1–2% of NSCLC patients [70]. According to NGS, more than 80% of the tumor samples had *RET* variants in combination with *TP53* alteration and changes in the PI3K pathway, MAPK effectors, and other tyrosine kinase families [71]. *RET* variants are believed to be crucial in the treatment of NSCLC, and *RET* fusions are considered to be a key cause of EGFR-TKI resistance in 5% of osimertinib-resistant patients [72].

An NGS-based Guardant360 (G360; Guardant Health) study identified *KIF5B-RET* fusions that are highly specific to NSCLC, whereas non-*KIF5B-RET* fusions contributed to anti-EGFR therapy resistance [73]. To date, many studies have shown that RET activity is inhibited by several FDA-approved multikinase inhibitors including vandetanib, lenvatinib, sunitinib, alectinib, sorafenib, ponatinib, nintedanib, and regorafenib. Specifically, cabozantinib and vandetanib have been added to the ASCO guidelines for the treatment of RET-positive patients [74]. KIF5B-RET-positive patients had a positive response to cabozantinib, with significant clinical improvement occurring quickly [75,76]. A liquid biopsy study in NSCLC patients with the *KIF5B-RET* variant revealed the development of *RET* G810R, G810S, and G810C variants responsible for clinical resistance [77].

Non-invasive cfDNA screening using the NGS technique provides information on *RET* rearrangements in NSCLC and aids in the identification of potential targeted therapeutics required to improve patient outcomes.

### 3.4. BRAF/MEK Inhibitors

*BRAF* variants have been found in 2% to 3% of NSCLC patients, resulting in the activation of downstream pathways facilitating cell proliferation and survival [78,79]. Various BRAF inhibitors are available, such as vemurafenib and dabrafenib, for the clinical treatment of BRAF/MEK-positive NSCLC patients [80]. These patients receiving BRAF inhibitor treatment had response rates of 42% and 33%, whereas the combination of dabrafenib and trametinib exhibited a response rate of 64%, making it a successful combination regimen that has been approved by the FDA for clinical trials [60,81,82,83]. The majority of encouraging results are only temporary, as most patients develop resistance to BRAF inhibitors within a few months. Currently, a lack of knowledge in the molecular makeup of NSCLC patients treated with BRAF-targeted therapies (BRAF-TT) has hampered the development of future targeted therapeutic strategies. In a study, CastPCR identified 6.5% of NSCLC patients (7/107) positive with the plasma *BRAF* variant, where the sensitivity, specificity, and concordance for the *BRAF* variant were 28.6%, 93.0%, and 88.8%, respectively [84]. ctDNA in blood samples from 208 patients detected the *BRAF* V600E variant in 74% of BRAF-TT-naive patients, suggesting a mechanism of resistance to BRAF-TT or *BRAF/MEK* combination therapy. Finally, investigations have demonstrated that genomic ctDNA profiling from a blood sample can give insight into the biology of BRAF mutants in NSCLC patients, as well as predict what mechanism is involved [85].

### 3.5. MET Inhibitors

MET is a receptor tyrosine kinase that phosphorylates HER3 in response to hepatocyte growth factor (HGF), in turn activating the PI3K pathway [86,87]. Several studies have demonstrated that *MET* amplification is responsible for 20% of EGFR-TKI-induced resistance [88,89]. Crizotinib is a MET inhibitor that selectively targets NSCLC tumors that harbor a *MET* exon 14 splice site [90]. Drug resistance has been reported for MET-TKIs, although relatively few studies have investigated the causes. In recent work, two newly acquired *MET* variants, Y1248H and D1246N, were identified in ctDNA, which could be a possible cause of resistance mechanism for type I MET-TKIs. This study shed light on the processes causing MET inhibitor resistance [91].

## 4. Prediction of Response to Immunotherapy

Immune checkpoint inhibitors have revolutionized the treatment of advanced NSCLC. However, it is clear that in the long term, only a small percentage of patients benefit from this approach. Due to the high expense of immunotherapy medications as well as the potential for long-term side effects, finding more accurate biomarkers is crucial for cost-effectiveness [92,93,94,95].

The FDA and ASCO approved tumor mutational burden (TMB) as a predictive biomarker for patients with lung cancer receiving immunotherapy [96]. The status of TMB in cancer patients can be determined by using whole-genome sequencing, whole-exome sequencing, or the targeted NGS of smaller sets of genes [97,98,99]. Many issues, such as tissue supply, have challenged the implication of TMB assay in clinical trials; however, this limitation can be partially overcome by analyzing TMB through ctDNA in blood [100].

In recent years, clinicians have paid close attention to the function of the blood tumor mutational burden (bTMB) as a biomarker for immunotherapy. Gandara et al. conducted the study on 1000 patients and demonstrated that TMB based on cfDNA measured with a novel blood-based sequencing assay rather than a tumor-tissue biopsy can predict the clinical benefit from treatment with anti-PDL-1 drug atezolizumab therapy [101].

A study on NSCLC patients treated with anti-programmed cell death protein 1 (PD-1)/programmed cell death ligand 1 (PD-L1) found that patients with a higher blood TMB (bTMB) had a shorter PFS and OS [102]. In follow-up studies, for a POPLAR and OAK cohort following immunotherapy, a Low Allele Frequency LAF-bTMB was highly related to favorable OS, PFS, and ORR, which was subsequently verified in the National Cancer Center (NCC) cohort [103]. Based on the findings, ctDNA appears to be a promising biomarker for immunotherapy prediction.

## 5. Prediction of Response to Chemotherapy

Cytotoxic chemotherapy continues to play a major part in the treatment of NSCLC. However, chemotherapy alone does not necessarily play a significant role in potentially curative NSCLC treatment. In certain trials, adjuvant chemotherapy and neoadjuvant therapy have been found to increase survival in excised stages, with a 5–10% survival benefit [104,105]. Chemotherapy prediction plays an important part in personalized medicine, but current biomarkers fail to forecast treatment effects.

Higher baseline cfDNA was associated with a considerably worse OS and a doubled risk of death in NSCLC patients treated with first-line chemotherapy [106]. According to the NCCN guidelines, platinum-based chemotherapy is recommended as the first-line chemotherapy strategy for advanced NSCLC. Next-generation sequencing using a BGI Oseq-ctDNA panel revealed somatic variants such as *EGFR* (L858R) and *KRAS* (G12C) and demonstrated a reduction in mutational load after platinum-based treatment. These findings indicated the potential predictive impact of mutation burden and a subset of gene variants in chemotherapy and precision treatment prediction [107].

## 6. Monitoring of the Disease Course and Effect of the Therapy

Liquid biopsy has gained wide attention in medicine and science recently because of the possibility of a real-time monitoring approach to assess the therapeutic response and resistance to therapy of each patient. CTC, exosomes, and ctDNA from plasma are commonly utilized for treatment monitoring, but with the advances in NGS, ctDNA has gained unique importance [108].

### 6.1. EGFR Inhibitors

The presence of *EGFR* variants in plasma DNA provides an ability to detect the *EGFR* variants and thus monitor the course of lung cancer treatment. The NGS-based study detected EGFR variants in TKI-treated lung cancer tissue, suggesting that tumor-specific variants in ctDNA can be a monitoring marker of TKI treatment in NSCLC patients [109]. Likewise, in the treatment cycle for patients, the variant status of T790M–*EGFR* in ctDNA was analyzed by Cobas, ddPCR, and NGS. The reduction in T790M–*EGFR* mutant fractions during treatment affected the clinical outcome of patients receiving treatment with osimertinib [110]. Another study used a ddPCR and NGS-based approach and identified allele frequency in 30 somatic variants in the cfDNA of afatinib-treated patients [111].

### 6.2. ALK Inhibitors

Dietz et al. employed cfDNA to perform copy number variation (CNV) profiling and ALK+ targeted panel sequencing on NSCLC patients who were undergoing TKI therapy. Shallow whole-genome sequencing (sWGS) detected CNV in 18% of the samples, including potentially druggable targets such as EGFR, ERBB2, and MET regions. The analysis revealed that combining copy number variants and targeted variant profiling could improve ALK+ NSCLC monitoring [112]. The use of this technology in the future could result in the serial monitoring of genetic biomarkers for NSCLC patients treated with ALK TKIs.

### 6.3. Immunotherapy (Anti-PD1/PDL1)

Since the arrival of selective therapies, immunotherapy that specifically targets the PD-1/PD-L1 checkpoint has been a promising strategy in combating NSCLC [113]. These immune checkpoint inhibitors cause tumor reactions that are distinct from those associated with a variety of other anti-cancer medications. However, there is a substantial need for finding new biomarkers to quickly monitor the course of immunotherapy on NSCLC patients [114]. A blood-based biomarker with rapid kinetics could be used to assess immunotherapy responses. Recent research has found that a decrease in ctDNA levels could be considered an early predictor of longer survival in NSCLC patients treated with immune checkpoint inhibitors. Monitoring the levels of *KRAS* variant in ctDNA, on the other hand, may allow the distinction between pseudo-progressive and progressive patients [115,116]. Anagnostou et al. evaluated ctDNA levels in metastatic lung cancer patients and found that patients responding to clinical treatment had a total decrease in ctDNA levels after therapy, whereas non-responders had no increase in ctDNA levels. The ctDNA level was initially lowered in patients who developed treatment resistance, followed by an increase in ctDNA levels. Furthermore, ctDNA dynamics predicted the pathologic response to immunotherapy by assessing the growth of T cells [117]. These trials provide a way to monitor the outcome of immunotherapy in NSCLC patients, which might lead to the advancement of customized immune-mediated treatments.

### 6.4. Chemotherapy

In recent years, there has been a large increase in the awareness of ctDNA as an indicator and biomarker for the real-time monitoring of the treatment response and survival in NSCLC patients. Jiang et al. detected alterations in 17 genes—*ALK*, *BCL2*, *BRAF*, *CD74*, *CDKN2A*, *EML4*, *GSTP1*, *KIF5B*, *KRAS*, *MLH1*, *MTHFR*, *NRAS*, *RRM1*, *PIK3CA*, *SLC34A2*, *XPC*, and *XRCC1*—after platinum-based doublet chemotherapy. This indicated that the mutational landscape in cfDNA has the potential to predict treatment response in NSCLC. Furthermore, NSCLC patients with low *TP53* molecular mutational burden strongly reacted to the platinum-based doublet therapy and had a longer free survival compared to those with a high mutational burden. This could be valuable information for monitoring chemotherapy in NSCLC patients [118]. The detection of genetic changes in cfDNA, such as *EGFR*, *KRAS*, and *BRAF* variants, helps to improve the monitoring of chemotherapy or targeted therapy in NSCLC patients. The *KRAS* variant is seen in around 30% of NSCLC patients and is associated with a reduced response to treatment. According to a study by Guibert et al., the presence of a *KRAS* variant was linked to a poor response to chemotherapy or targeted therapy. Moreover, the monitoring of ctDNA for the *KRAS* variant revealed that the ctDNA *KRAS* variant level throughout targeted or conventional therapy was linked to treatment response. Despite the limited sample size, the study revealed that the detection of variants in ctDNA through ddPCR is far more sensitive and stable when used as a marker for monitoring the course of chemotherapy [119].

### 6.5. Radiotherapy

Radiation therapy is being used in around 45% of I-III NSCLC patients; however, a limited number of studies conducted a deep analysis of the influence of irradiation on cfDNA in NSCLC [120]. A pilot study analyzed a cohort of stage II–III NSCLC patients during the first week of radical radiotherapy. After 72 h, there was a substantial increase in ctDNA levels; however, at the 7-day timepoint, there was a non-significant drop in ctDNA levels. The study revealed the possibility of using ctDNA as a biomarker in a small number of patients, but larger studies with a wider range of time points and disease stages need to be conducted to assess ctDNA as a radiation-monitoring biomarker [121].

## 7. Further Use of ctDNA in NSCLC

### 7.1. NSCLC Diagnosis

For decades, X-rays and computed tomography (CT) have been widely used for NSCLC screening and diagnosis, but these methods are insufficiently sensitive for early detection. Positron emission tomography (PET) imaging has demonstrated a high sensitivity and potential for detecting NSCLC, but at a high cost. Despite many studies on liquid biopsy in lung cancer, there are only limited data on ctDNA as a screening tool for NSCLC.

Due to the biological background of ctDNA in the early stages of cancer, cancer screening tests where even ultrasensitive methods of detection are involved are still challenging. The main reasons are the low level of ctDNA and the limited number of somatic variants, making it difficult to discriminate tumors from controls.

Currently developed multi-cancer early detection (MCED) tests with the aim to obtain FDA approval are based on combining blood-based cancer biomarkers using both protein and genetic markers. A study by Cohen et al., in which the CancerSEEK test was used on a group of 1005 cancer patients including 104 lung cancer patients (stage I–III), showed sensitivity in nearly 60% of lung cancer patients. This blood-based test combines the ctDNA genotyping of 16 cancer-related genes and 41 potential protein biomarkers [122]. A CE-IVD test, Epi proLung (Epigenomics AG, Berlin, Germany), focuses on the detection of the ctDNA methylation profile of genes such as *SHOX2* and *PTGER4* [123].

Once the tumor is detected, tissue biopsy is the gold standard for analyzing various morphological and genetic changes and for classification into histological subtypes. Histologically, NSCLC can be classified into three main subtypes: squamous cell carcinoma (25% of lung cancers), adenocarcinoma (40% of lung cancers), and large cell carcinoma (10% of lung cancers). It is vital to make an early diagnosis and determine the extent of the disease.

Liquid biopsy is a less demanding approach. Due to the biological features of tumor tissue, proteins and nucleic acids with a specific signature could be detected in the bloodstream. The analysis of ctDNA for the methylation profile of certain genes appears to be effective for early diagnosis. A study on NSCLC patients found methylation in seven genes (*P16*, *RASSF1A*, *APC*, *RAR*, *DAPK*, *CDH13*, and *MGMT*) and suggested *RASSF1A* and *RAR* as significant biomarkers with high sensitivity and specificity. A methylation study approach adapted by Yang et al., using quantitative methylation-specific polymerase chain reaction (QMSP) technology, also detected methylation in *RASSF1A*, *CDKN2A*, and *DLEC1*, which distinguished early and benign lesions in lung cancer. Moreover, when compared to pathological regression analysis results, methylation in ctDNA detected early lung cancer in 70% of patients with a specificity of 95–100% [124]. Ooki et al. established a methylation panel of six different genes (*CDO1*, *HOXA9*, *AJAP1*, *PTGDR*, *UNCX*, and *MARCH11*) assessed in blood serum with the potential for early NSCLC detection. They demonstrated that the aberrant promoter methylation of these genes had similar sensitivity and superior specificity when compared to the CT screening of IA-stage lung adenocarcinoma patients [125].

Furthermore, specific variants in ctDNA have been tested for usage in the early diagnostics of NSCLC. Epidermal growth factor receptor (*EGFR*) variants are considered to be of high significance in NSCLC. Passiglia et al. published a meta-analysis investigating the diagnostic accuracy of the *EGFR*-T790M variant detected in ctDNA in patients with advanced NSCLC. The study found a pooled sensitivity of 0.67. This result was similar to the one reported for the detection of this particular variant among NSCLC patients [126].

### 7.2. Assessment of Tumor Burden

The total mass of tumor tissue, including the tumor cells distributed in bone marrow in patients with cancer, is referred to as a tumor burden [127]. Tumor burden surveillance is important in all phases of cancer management to prevent metastasis and recurrence [128]. Clinically, tumor burden can be characterized by the number of metastatic lesions, location of metastases, tumor size, and high lactate dehydrogenase (LDH) content [129]. The significance of tumor burden in cancer diagnosis has been investigated, but the methods applied for assessment produce inconclusive results [130]. A rapidly proliferating tumor, larger than 4 cm in maximal diameter, tends to outgrow the blood supply, inducing necrosis in the core of the tumor. This phenomenon releases fragments of tumor DNA into the bloodstream, relating mutated ctDNA to tumor burden [131,132]. Considering this concept, new approaches are being developed to assess tumor burden using ctDNA. The concentration of plasma mutant *EGFR* T970M in ctDNA was detected using a ddPCR approach and was found to be significantly correlated with the number of metastatic sites, the number of lesions, and the sum of measurable lesions’ diameters. This demonstrated the tremendous potential of *EGFR* variants for use in assessing tumor burden in NSCLC patients [133,134].

Studies demonstrate that assessing tumor burden alone is not a perfect predictor, but the tumor mutation burden appears to be a steady indicator due to its intrinsic nature. Tumor mutational burden (TMB) is broadly defined as the number of various somatic variants present in the tumor. TMB is closely linked to variants in DNA replication pathway genes such as *POLD1* and *POLE*, which can be measured by whole exome sequencing (WES) or comprehensive genomic profiling (CGP, or gene panels sequencing) [135].

### 7.3. Estimation of Prognosis

The most desirable method to assess the prognosis is one that would allow the determination of the extent of the disease, i.e., the identification of the disease stages that could vary depending on injuries, disease, age, sex, race, and received treatment. Results have been published that show the correlation not only between NSCLC tissue variants and prognosis but also between ctDNA variants and prognosis, implying that ctDNA could be a valuable tool for estimating prognosis or predicting a patient’s survival [136,137]. A study by Jia et al. demonstrated conclusively that ctDNA was detected in the initial stages of NSCLC patients, and the quantities of ctDNA were 1.4-fold higher in patients with bone metastasis [138]. Michaelidou et al. recently supported this study by detecting the *KRAS* G12/G13 variant in the plasma ctDNA of NSCLC patients in first-line systemic treatment, which was significantly associated with poor patient disease outcomes, in terms of PFS and OS, serving as an independent biomarker of unfavorable prognosis in NSCLC patients [139]. Yanagita et al. assessed plasma mutant *EGFR* (ex19del, L858R, and T790M) in cfDNA using a ddPCR approach, and it was found that there was a significant relationship between a high level of cfDNA (≥55 *EGFR* variant copies/mL) and shorter PFS [134].

Allele frequency heterogeneity (AFH) detected in ctDNA helped in predicting prognosis in advanced NSCLC patients. An NSCLC cohort positive for AFH in ctDNA significantly correlated with unfavorable OS in advanced NSCLC patients [140]. Song et al. performed a large study exploring the genomic landscape in 1336 Chinese patients. The study found that higher ctDNA abundance and variant number were significantly related to shorter OS. Moreover, during treatment, ctDNA clearance and variant clearance (rate of decrease) was significantly associated with longer PFS and OS, making it a useful prognostic marker for a wide variety of treatment methods [141].

### 7.4. Prediction and Detection of Recurrence after Tumor Resection

The factor responsible for the postoperative recurrence of tumors can be identified by two methods: clinical parameters (TNM classification) and molecular profiling. In the early stages of the disease, the prediction of relapse in patients undergoing curative resection is useful for decisions about adjuvant treatment. There are methods for the prediction of relapse in patients undergoing tumor resection based on variant analysis and gene expression in tissue; however, ctDNA could provide this information. Postoperative recurrence has been reported in one-third of early-stage NSCLC patients, and recent studies show that ctDNA-based molecular profiling can emerge as a non-invasive biomarker tool to provide better disease prognosis [4]. However, no specific standards have been determined yet, concerning the ideal time for ctDNA detection as postoperative surveillance in early-stage cancer. The half-life of ctDNA after surgery was found to be 114 min in stage-IV colorectal cancer patients [142]. For the first time, perioperative dynamic changes in ctDNA confirmed that plasma taken from patients who had curative-intent lung resections after three days was better than plasma collected after 1 day or after 30 days [143]. Furthermore, variants in ctDNA were reduced 2 days after surgery by up to 91.7%, confirming a 2- to 3-day time point as a standard for postoperative lung cancer surveillance. This makes it convenient for clinical decision-making [144]. A high level of variant allele frequency (VAF) in preoperative ctDNA predicted lymph node metastasis in patients with resectable NSCLC [145]. As per the NCCN guidelines, NSCLC patients undergoing radical surgery usually require follow-up with CT contrast scans every 6 months for the first 2–3 years for monitoring the possibility of recurrent disease [146]. However, the combined results of five studies, involving 351 NSCLC patients, confirmed that CTCs (HR, 3.37; *p* = 0.001) and ctDNA (HR, 8.15; *p* = 0.002) can predict postoperative recurrent disease in NSCLC patients in one to two years after the primary surgery [147]. 

In early-stage resected NSCLC, the quantification of plasma ctDNA can be used to assess minimal residual disease (MRD) and for the early detection of disease recurrence. In the study of Chaudhuri et al., the detection of ctDNA preceded radiographic progression in 72% of patients [148]. Recently, clinical trials have been conducted on patients with detected ctDNA, before and after surgery; for instance, “Evaluation Perioperative Dynamic Changes in ctDNA from Patients of Non-Small-Cell Lung Cancer Following Resection for Relapse Prediction” (ClinicalTrials.gov Identifier: NCT04238130) and “Role of Circulating Tumor DNA (ctDNA) From Liquid Biopsy in Early Stage NSCLC Resected Lung Tumor Investigation” (ClinicalTrials.gov Identifier: NCT03553550). These data suggest that ctDNA could be a significant marker for the prediction and detection of disease recurrence in patients who had tumor resection surgeries.

### 7.5. ctDNA Detection from Cerebrospinal Fluid

It was shown that ctDNA derived from brain tumors is more abundantly present in CSF than in blood plasma [149]. Moreover, the genomic alterations in metastases to the brain differ from those of primary tumors [150]. Around 7% of NSCLC patients had brain metastases at the time of diagnosis, and 20–40% of patients acquire brain metastases at some point throughout their disease [151]. The presence of ctDNA in cerebrospinal fluid (CSF) has been proved in recent research. The examination of tumor-specific genes in CSF-ctDNA provides a platform for identifying brain metastases in NSCLC patients. The next-generation sequencing and ddPCR identified EGFR variants in CSF-ctDNA to a larger extent in comparison to the blood ctDNA and CTCs of patients with brain and leptomeningeal metastases. Moreover, *KIT*, *PIK3CA*, *TP53*, *SMAD4*, *ATM*, *SMARCB1*, *PTEN*, *FLT3*, *GNAS*, *STK11*, *MET*, *CTNNB1*, *APC*, *FBXW7*, *ERBB4*, and *KDR* (all > 10%) were the most mutated genes in CSF-ctDNA [152,153]. 

NSCLC patients who have developed CNS metastasis are typically treated with osimertinib [154]. Zheng et al. performed the genotyping of CSF-ctDNA before the first dosing of osimertinib and after tumor progression. Variants in *EGFR* C797S, *TP53*, and *RB1* were detected at the time of the development of osimertinib resistance, suggesting a mechanism for osimertinib resistance [155].

The published results indicate that genetic analysis by the liquid biopsy of CSF can help to identify NSCLC brain metastases and the resistance mechanisms of clinically accessible treatments.

### 7.6. ctDNA Detection from Urine

Two decades ago, it was shown that DNA present in systemic circulation is able to pass through the renal glomerular filtration membrane and be excreted into urine [156]. Urine sampling provides a non-invasive source of ctDNA from cancer patients, and daily urine collection is easily performed. Studies based on patient-matched tissue, plasma, and urine indicate the concordance of DNA variant status and the comparable sensitivities of these three biospecimens [157,158]. 

Results show that urinary DNA measurements can potentially be useful for disease monitoring, even of minimal residual disease in NSCLC [159]. In 2017, Husain et al. focused on the monitoring of early tumor responses to third-generation TKIs by ctDNA in urine. He described patients responding to targeted therapy, involving a kinetic increase in the number of copies of ctDNA immediately after therapy, for the first time. The subsequent rapid loss of mutant *EGFR* ctDNA in urine after the first weeks of treatment was associated with the assessment of the radiographic response [160]. Based on the case reports, Tchekmedyian et al. concluded that the longitudinal monitoring of ctDNA *EGFR* variant burden from urine correlates with patients’ response to EGFR-TKIs [161]. Unfortunately, to date, not enough studies have been conducted to really evaluate the relevance and clinical usability of urine ctDNA for NSCLC prediction.

## 8. Conclusions

The management of oncology patients is based on imaging methods, tissue biopsies, and histopathology, when these approaches could be marked as irreplaceable without any doubt. However, there are some parts of patient management that could be improved or supported by the approach of liquid biopsy.

The liquid biopsy approach allows for repeated examinations without a greater burden on the patient. As ctDNA reflects the changing variant profile of the tumor tissue during the course of the disease, it can well address the requirements of targeted therapy. If there is a need to identify specific genetic variants, the digital PCR-based approach will be ideal due to its high sensitivity and low cost. NGS-based approaches are currently available for the identification of a broader range of variants. It can be expected that, as the spectrum of targeted therapeutics expands, these methodologies will become a part of recommendations for the management of NSCLC patients.

## Data Availability

Not applicable.
